# Small Discrete Pre‐Potential‐Like Electrogram Recorded by a Lattice‐Tip Catheter at the Initiation of Atrial Fibrillation

**DOI:** 10.1002/joa3.70501

**Published:** 2026-10-14

**Authors:** Yasuharu Matsunaga‐Lee, Yasuyuki Egami, Masamichi Yano, Masami Nishino

**Affiliations:** ^1^ Division of Cardiology Osaka Rosai Hospital Osaka Japan

## Abstract

At the origin of the non‐PV trigger, a small discrete pre‐potential‐like electrogram was recorded using a lattice‐tip catheter after 5‐mm catheter repositioning, preceding the potential at the previous site by 56 ms.

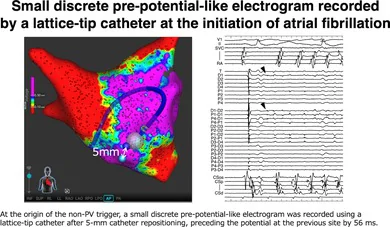

A woman in her 70s underwent repeat catheter ablation for paroxysmal atrial fibrillation (AF) after an initial procedure consisting of pulmonary vein (PV) isolation and non‐PV trigger ablation at the left atrial septum. During the repeat procedure, a 10‐μg isoproterenol bolus induced immediate AF recurrence. During AF initiation, the coronary sinus activation sequence was proximal‐to‐distal, and the right atrial lateral wall catheter was activated later than the coronary sinus catheter, suggesting a septal origin of the trigger. Using the Affera Prism‐2 mapping system (Medtronic), a lattice‐tip catheter (Sphere‐9; Medtronic) was positioned at the presumed site of origin (Figure 1A, light blue tag). At AF initiation, the earliest potentials recorded by the Sphere‐9 catheter (Figure 1C, white arrowheads) were earlier than the electrograms recorded by the other catheters placed in the right atrium and coronary sinus. However, the degree of prematurity was limited. The catheter was moved 5 mm superiorly (Figure 1B, blue tag), and a discrete pre‐potential‐like electrogram was identified (Figure 1D, black arrowheads). The potentials were recorded in both Close‐Unipolar and bipolar recordings using an external EP laboratory recording system with a 30–250 Hz band‐pass filter and preceded those recorded at the previous site by 56 ms. During sinus rhythm, the difference in local activation time between the two sites (light blue and blue tags) was 8 ms, and the electrogram at the second catheter site (D1) had an amplitude of 2.01 mV without apparent fractionation (Figure 2). At the onset of AF, a discrete pre‐potential‐like electrogram (peak‐to‐peak amplitude, 0.13 mV) was recorded with a coupling interval of 206 ms from the preceding sinus beat (Figure 1D). A similar small potential was also observed during the subsequent beat after AF initiation (Figure 1D). Because the trigger originated from the atrial septum and was considered to be in proximity to the atrioventricular node and His bundle (Figure 3), the pulsed field ablation application duration was empirically shortened to 2 s to minimize the potential effect on the conduction system. After a 20‐min waiting period, AF could not be induced despite repeated 20‐μg isoproterenol boluses. At 2‐month follow‐up, the patient remained free from palpitations, whereas palpitations were already present at the 1‐month follow‐up after each of the previous two ablation procedures.

**FIGURE 1:**
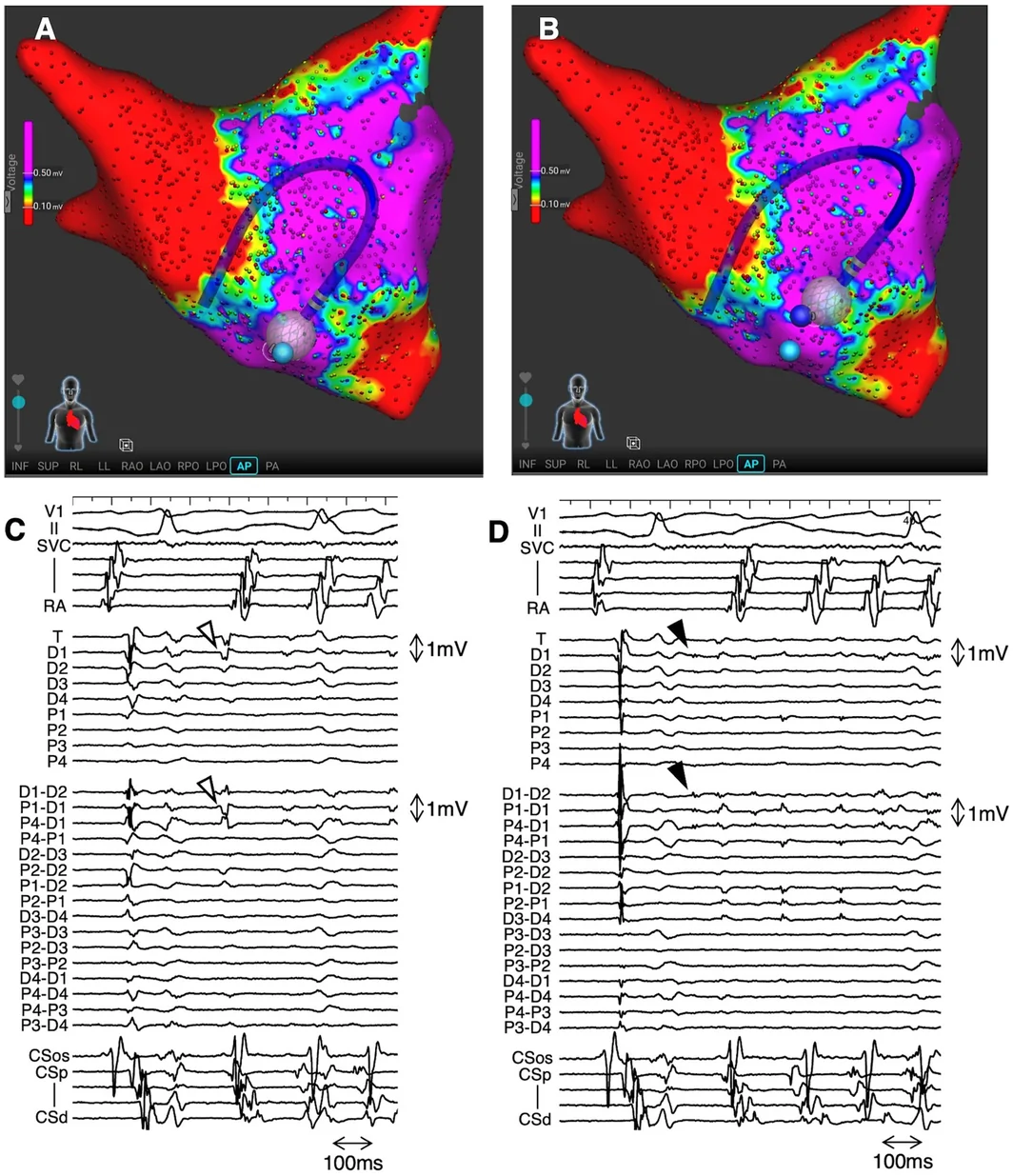
Identification of a small discrete pre‐potential‐like electrogram at AF initiation. (A) Initial position of the lattice‐tip catheter (light blue tag). (B) Catheter position after moving 5 mm superiorly (blue tag). (C) Intracardiac electrograms at the initial site (A). White arrowheads indicate the earliest potentials recorded by the lattice‐tip catheter. (D) Intracardiac electrograms at the second site (B). Black arrowheads indicate the discrete pre‐potential‐like electrogram, which preceded the potential at the initial site by 56 ms. A discrete pre‐potential‐like electrogram (black arrowhead) with a peak‐to‐peak amplitude of 0.13 mV was recorded by the Close‐Unipolar electrode (D1), with a coupling interval of 206 ms from the preceding sinus beat. AF, atrial fibrillation; PV, pulmonary vein.

**FIGURE 2:**
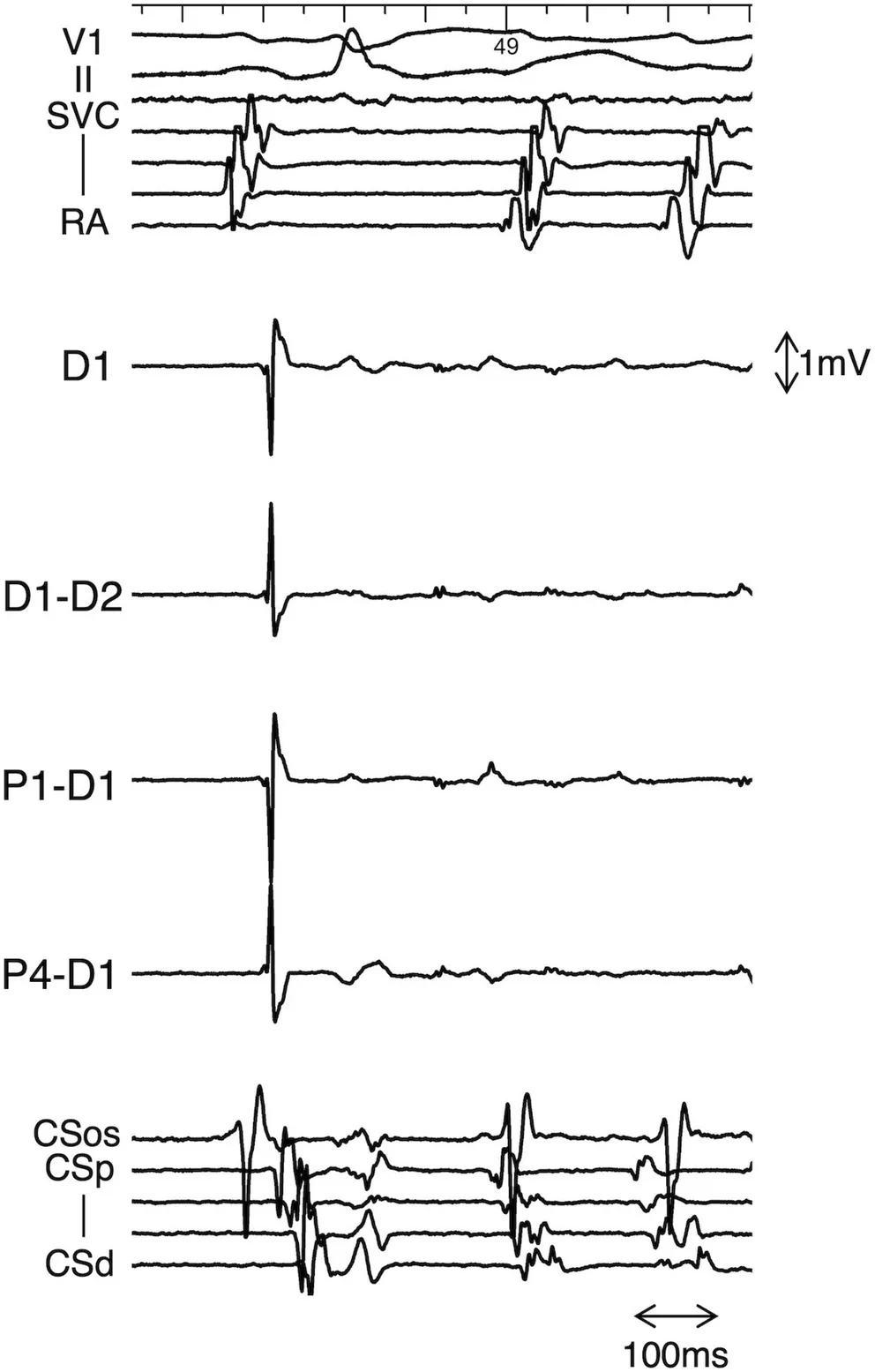
Intracardiac electrograms recorded at the catheter position shown in Figure 1B. No apparent electrogram fractionation was observed during sinus rhythm.

**FIGURE 3:**
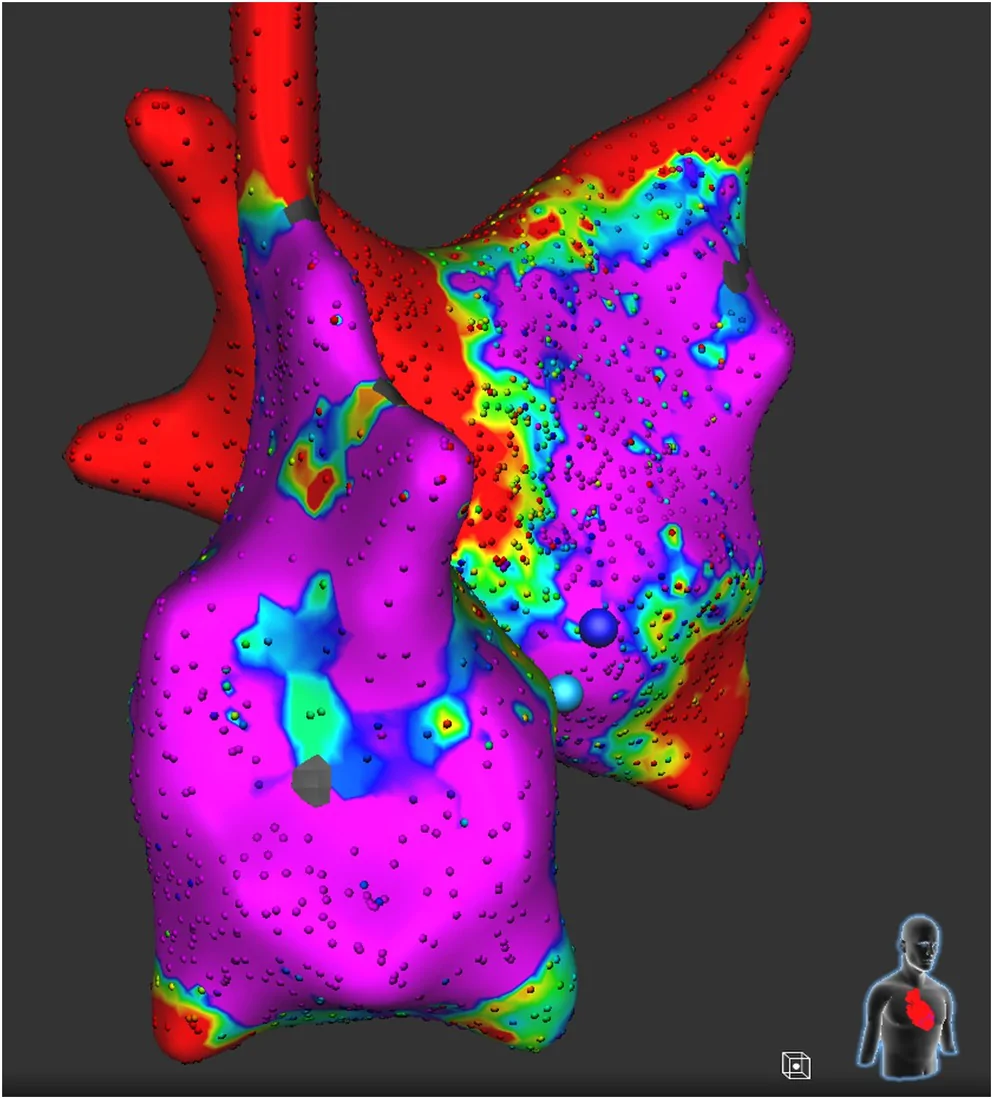
Anatomical relationship between the trigger site and the right and left atria. Right and left atrial voltage maps in the right anterior oblique view. The light blue and blue tags indicate the first and second catheter positions, respectively. Based on their anatomical location, the trigger site was considered to be in proximity to the atrioventricular conduction system. The His bundle electrogram was not tagged. Mapping of the trigger from the right atrial side was not performed. The right atrial voltage map was not completely acquired in some regions.

To identify the origin of a non‐PV trigger, multipolar catheters are used to map a broad endocardial surface, allowing simultaneous comparison of local activation timing within the region of interest [1, 2]. Because the lattice‐tip catheter has a sphere‐shaped configuration, maintaining adequate contact over a broad endocardial surface may be challenging, potentially increasing the risk of missing small electrograms. In the present case, step‐by‐step movement of the catheter revealed a marked change in local prematurity, as demonstrated by the 56‐ms difference between two sites only 5 mm apart. Such a change in local prematurity may help guide identification of the precise trigger origin. Further studies are needed to determine how much local prematurity or how short a coupling interval from the preceding sinus beat is useful for identifying the precise origin. The marked difference in local prematurity became apparent only at AF initiation, whereas the difference in local activation time between the two sites was 8 ms during sinus rhythm. In our previous study, we discussed the possibility that conduction from a small trigger myocardium to the surrounding myocardium may be delayed at the initiation of AF because of source‐sink mismatch [1]. These findings suggest that the electrophysiological characteristics of some non‐PV triggers may become apparent only at AF initiation.

We also reported that small electrograms recorded using bipolar electrodes were a key finding for identifying the precise origin of non‐PV triggers. Moreover, we discussed the possibility that small potentials arising from diseased tissue may be masked by surrounding larger potentials during sinus rhythm and could be involved in AF initiation through a reentrant mechanism [1]. In the present case, a similar small potential was observed during the subsequent beat after AF initiation, suggesting local reentry as one possible mechanism, although the precise mechanism could not be determined. In the present case, a discrete pre‐potential‐like electrogram with a short coupling interval from the preceding sinus beat was successfully recorded using the Close‐Unipolar electrode of a lattice‐tip catheter. Because bipolar electrogram amplitude can be affected by electrode geometry and orientation relative to the direction of wave propagation [3], bipolar blindness could have contributed to the small amplitude of the AF‐initiating potentials in the previous report. However, the persistence of a low‐amplitude electrogram despite Close‐Unipolar recording suggests that these small electrograms may not be explained solely by bipolar blindness.

This case highlights that careful inspection of local electrograms combined with step‐by‐step catheter movement may help identify the precise origin of a non‐PV trigger even when using a sphere‐shaped catheter.

## Ethics Statement

Institutional review board: Osaka Rosai Hospital 2026‐85.

## Conflicts of Interest

The authors declare no conflicts of interest.

## Data Availability

The data that support the findings of this study are available from the corresponding author upon reasonable request.
